# Computational Insights into Reproductive Toxicity: Clustering, Mechanism Analysis, and Predictive Models

**DOI:** 10.3390/ijms25147978

**Published:** 2024-07-22

**Authors:** Huizi Cui, Qizheng He, Wannan Li, Yuying Duan, Weiwei Han

**Affiliations:** 1 Key Laboratory for Molecular Enzymology and Engineering of Ministry of Education, School of Life Sciences, Jilin University, Changchun 130012, China; hzcui23@mails.jlu.edu.cn (H.C.); heqz9923@mails.jlu.edu.cn (Q.H.); liwannan@jlu.edu.cn (W.L.); 2 Edmond Henri Fischer Signal Transduction Laboratory, School of Life Sciences, Jilin University, Changchun 130012, China; 3 School of Economics and Management, Inner Mongolia University of Science and Technology, Baotou 014010, China

**Keywords:** reproductive toxicity, reverse docking, molecular descriptors, molecular fingerprints, machine learning

## Abstract

Reproductive toxicity poses significant risks to fertility and progeny health, making its identification in pharmaceutical compounds crucial. In this study, we conducted a comprehensive in silico investigation of reproductive toxic molecules, identifying three distinct categories represented by Dimethylhydantoin, Phenol, and Dicyclohexyl phthalate. Our analysis included physicochemical properties, target prediction, and KEGG and GO pathway analyses, revealing diverse and complex mechanisms of toxicity. Given the complexity of these mechanisms, traditional molecule-target research approaches proved insufficient. Support Vector Machines (SVMs) combined with molecular descriptors achieved an accuracy of 0.85 in the test dataset, while our custom deep learning model, integrating molecular SMILES and graphs, achieved an accuracy of 0.88 in the test dataset. These models effectively predicted reproductive toxicity, highlighting the potential of computational methods in pharmaceutical safety evaluation. Our study provides a robust framework for utilizing computational methods to enhance the safety evaluation of potential pharmaceutical compounds.

## 1. Introduction

**Figure 1:**
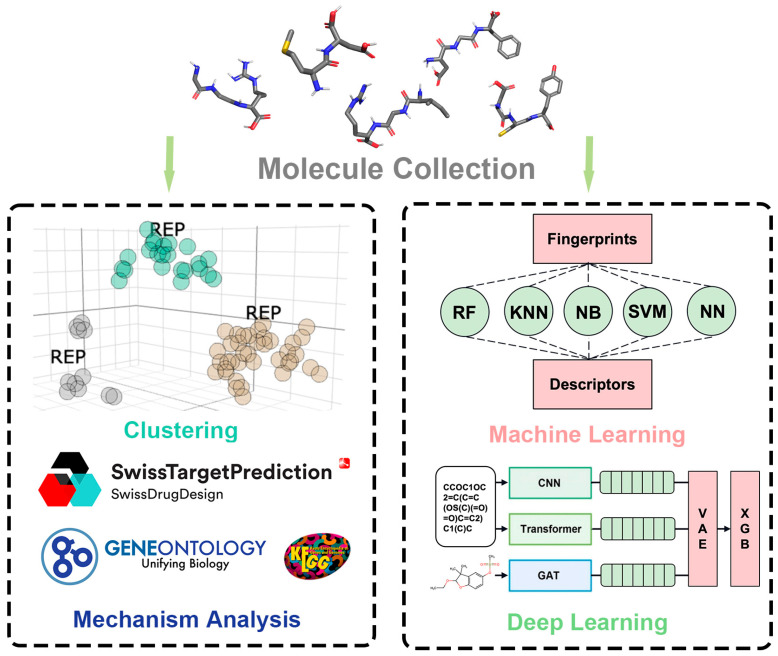
An overview of the study workflow.

The study of reproductive toxicity is critically important because it can significantly affect fertility and the health of offspring across several generations. It must be a priority to safeguard drugs and chemicals for the reproductive populace, particularly women of childbearing age and pregnant women. It is of the utmost importance to preclude the inadvertent introduction of substances that could precipitate birth defects or developmental anomalies that extend through generations. A large number of animal models have been developed to study reproductive toxicity [1,2]. However, the traditional in vivo methods for assessing reproductive toxicity, especially those that span multiple generations, are encumbered by their protracted timelines, often necessitating the traversal of at least three generations. Concurrently, these methodologies demand a substantial cohort of laboratory animals, thereby escalating the fiscal burden and engendering profound ethical and moral quandaries [3,4,5,6]. Given these constraints, it is impracticable to subject every pharmaceutical compound to such exhaustive in vivo multigenerational toxicity evaluations. This is where the advantages of computational methods for predicting reproductive toxicity become apparent. The prediction of reproductive toxicity by computer instead of animal in vivo experiments has the characteristics of a short test period, cost saving, and adherence to ethical norms regarding animal experiments. The conventional computer-based approach to toxicity prediction involves identifying the drug target and simulating the docking of the small-molecule drug with the target protein in the organism through the calculation of the drug structure, with the subsequent prediction of reproductive toxicity [7]. However, this methodology necessitates that the small-molecule drug has several limited targets. A chemical space analysis and clustering of small molecules with reproductive toxicity were conducted [8,9,10], along with reverse docking drug target prediction [11,12]. The analysis revealed that the number of uncertain targets for reproductive toxicity is too large for the target simulation approach to be a viable solution. The advent of machine learning (ML) presents a promising horizon for toxicity prognostication, offering non-invasive alternatives that may achieve high fidelity and expedience [13,14,15]. Despite the auspicious prospects of ML, the prognostication of reproductive toxicity stands as a formidable challenge. This complexity arises from the intricacies of biological mechanisms and the dearth of extensive and holistic data repositories. The paucity of data can engender overfitting, undermining the generalizability of predictive models, which is a significant issue in their development. So we constructed a predictive model that overcomes these impediments, focusing on reproductive toxicity. Our methodology is based on a hybrid approach that combines convolutional neural networks (CNNs) [16], Transformer, and graph attention networks (GATs) [17] for the extraction of salient features. These features are then reconstructed using a Variational Autoencoder (VAE) [18]. This innovative framework is designed to mitigate the limitations posed by diminutive datasets and to outperform conventional machine learning algorithms in terms of predictive accuracy. CNNs have demonstrated remarkable efficacy in image-based feature extraction across a spectrum of applications, including the prognostication of toxicity. Transformers, initially conceived for the parsing of natural language, have since manifested their adaptability in the domain of sequential data within machine learning. GATs, specialized in the processing of graph-structured data, have emerged as efficacious in capturing the sophisticated interplays within molecular compounds. VAEs, as generative models, offer an auspicious avenue for the reconstruction and denoising of features. The synthesis of these diverse neural network paradigms with a VAE for the specific purpose of multigenerational reproductive toxicity prognostication has not been extensively explored. Our methodology encompasses the training of CNNs and transformers on molecular sequence data, alongside the application of GATs to chemical structure imagery, to distill a comprehensive ensemble of features. Subsequently, these features are funneled through a VAE, which recalibrates the weights of the aforementioned models. Our hybrid model is subjected to rigorous training and validation on a modest dataset, which is then compared to the performance of traditional machine learning algorithms. This allows us to identify the merits and demerits of our proposed approach. It is our expectation that this scholarly pursuit will yield a pioneering hybrid ML model that is adept at identifying the multigenerational reproductive toxicity of substances. The juxtaposition with established algorithms will elucidate the comparative strengths and limitations of advanced neural network methodologies when contending with data scarcity. The findings of this study may herald a paradigm shift towards more efficacious and ethical strategies for toxicity assessment. The workflow of this study is shown in Figure 1.

## 2. Results and Discussion

### 2.1. Chemical Properties

**Figure 2:**
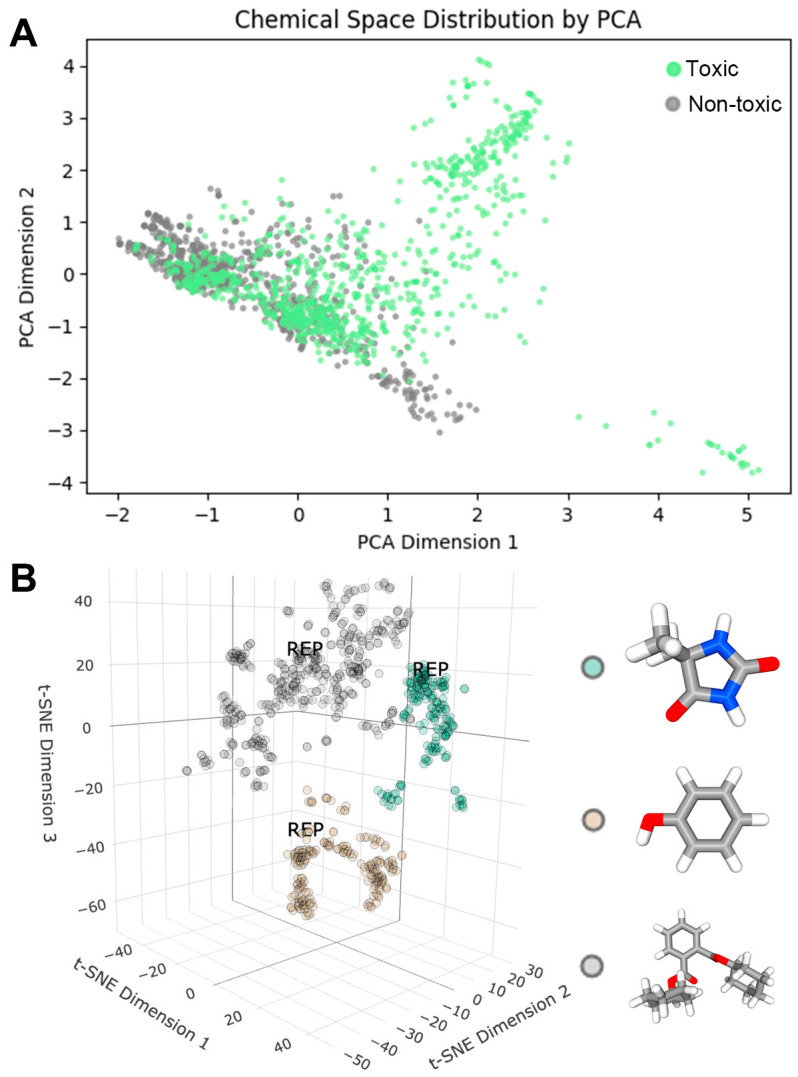
(**A**) Chemical space of reproductive toxic and non-toxic molecules. (**B**) Clustering results for reproductive toxic molecules.

We conducted various analyses on the collected molecules to understand their properties. Initially, we employed principal component analysis (PCA) [19] to explore the chemical space of reproductive toxic and non-toxic molecules, as illustrated in Figure 2A. It is evident that the distribution of non-toxic molecules in the chemical space is not as broad as that of toxic molecules, suggesting some common characteristics. However, because a large quantity of positive and negative data are consistent in the chemical space, it is impossible to distinguish between positive and negative data effectively by reducing the dimensions of the data, which is why we further explored machine learning and deep learning. However, the presence of multiple small clusters indicates that these molecules may induce reproductive toxicity through different mechanisms. Consequently, we performed a clustering analysis on the reproductive toxic molecules (Figure 2B), using hierarchical clustering based on MACCS molecular fingerprints [20]. Representative molecules for each cluster were identified through similarity analysis. The molecules were classified into three clusters: Cluster 1 represented by Dimethylhydantoin, Cluster 2 by Phenol, and Cluster 3 by Dicyclohexyl phthalate. The clustering results are presented in Table S1.

**Figure 3:**
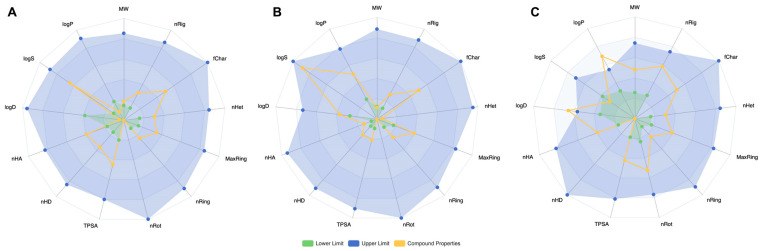
Physicochemical properties of three representative molecules. (**A**) Dimethylhydantoin. (**B**) Phenol. (**C**) Dicyclohexyl phthalate.

Next, we analyzed the physicochemical properties of the three representative molecules. Physicochemical properties are thermodynamically defined by a set of macroscopic quantities that are accessible to experimental measurements and related to the laws of statistical mechanics governing their microscopic parts. The radar charts in Figure 3 illustrate the properties of the representative molecules from each cluster. The parameters for each chart include molecular weight (MW), logP, logS, logD, the number of hydrogen bond donors (nHD) [21], the number of hydrogen bond acceptors (nHA) [22], topological polar surface area (TPSA) [23], the number of rotatable bonds (nRot) [24], the number of rings (nRing) [25], the maximum ring size (MaxRing), the number of heteroatoms (nHet), the proportion of aromatic rings (fChar) [26], and the number of rigid bonds (nRig). Overall, these compounds exhibit different performances across various parameters. Dimethylhydantoin and Dicyclohexyl phthalate have lower hydrophobicity and solubility with minimal rotational freedom, whereas Phenol shows moderate performance across most properties, especially in logS. Dicyclohexyl phthalate has the highest number of hydrogen bond acceptors. These distinct physicochemical properties reflect the success of our clustering, distinguishing the bio-toxic small molecules into three categories with representative molecules.

### 2.2. Target Prediction

**Figure 4:**
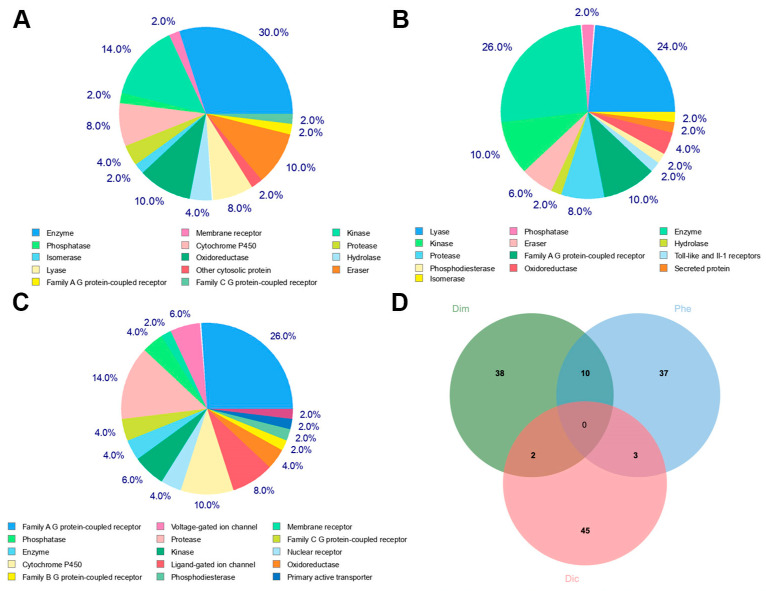
Distribution of top 50 targets for the three representative molecules. (**A**) Dimethylhydantoin. (**B**) Phenol. (**C**) Dicyclohexyl phthalate. (**D**) Intersection of targets.

We predicted the targets of the three representative molecules using SwissTarget Prediction [27] to analyze the molecular mechanisms underlying their toxicity. The targets for Dimethylhydantoin are listed in Table S2, those for Phenol in Table S3, and those for Dicyclohexyl phthalate in Table S4. We analyzed the top 50 targets, and the results are shown in Figure 4. The targets for Dimethylhydantoin include enzymes, phosphatases, and P450s, which may affect the intracellular environment. The targets for Phenol involve lyases and kinases, potentially impacting signaling pathways. The targets for Dicyclohexyl phthalate involve G-protein-coupled receptors, ion channels, membrane proteins, and nuclear receptors. The mechanisms of these three molecules are distinct, with minimal overlap in targets, highlighting the complexity of reproductive toxicity mechanisms.

**Figure 5:**
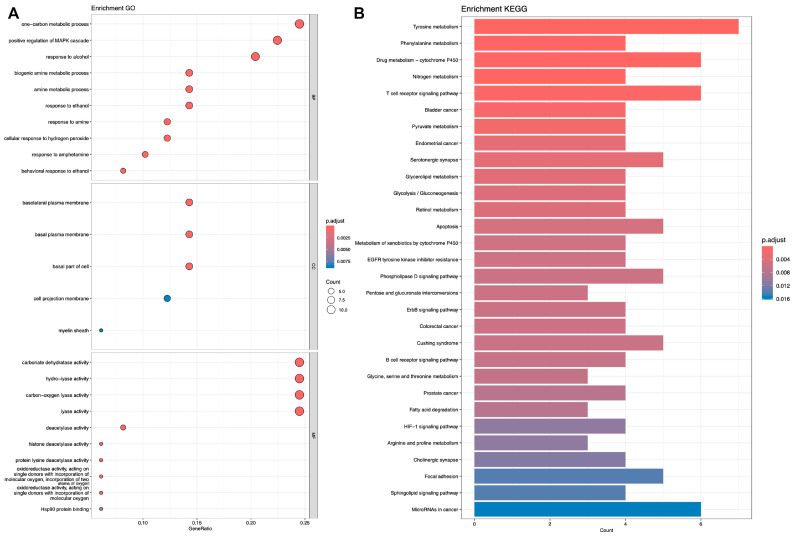
Analysis of representative molecule Dimethylhydantoin. (**A**) GO analysis. (**B**) KEGG analysis.

**Figure 6:**
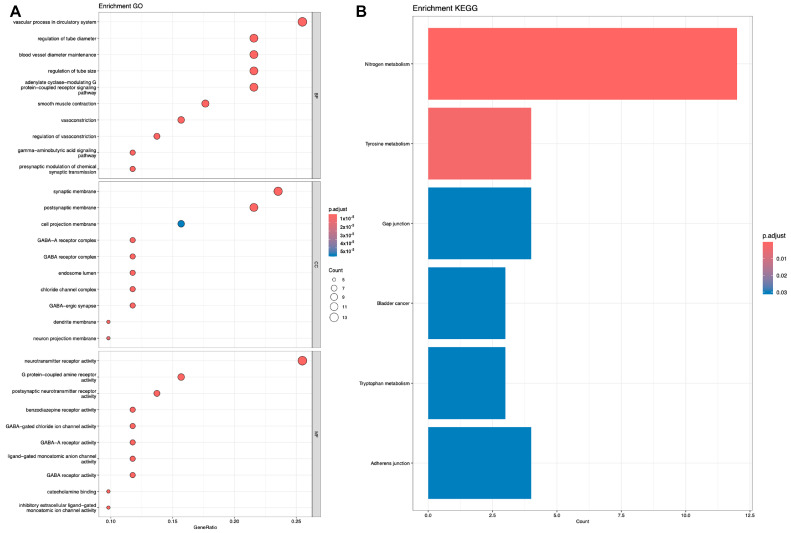
Analysis of representative molecule Phenol. (**A**) GO analysis. (**B**) KEGG analysis.

**Figure 7:**
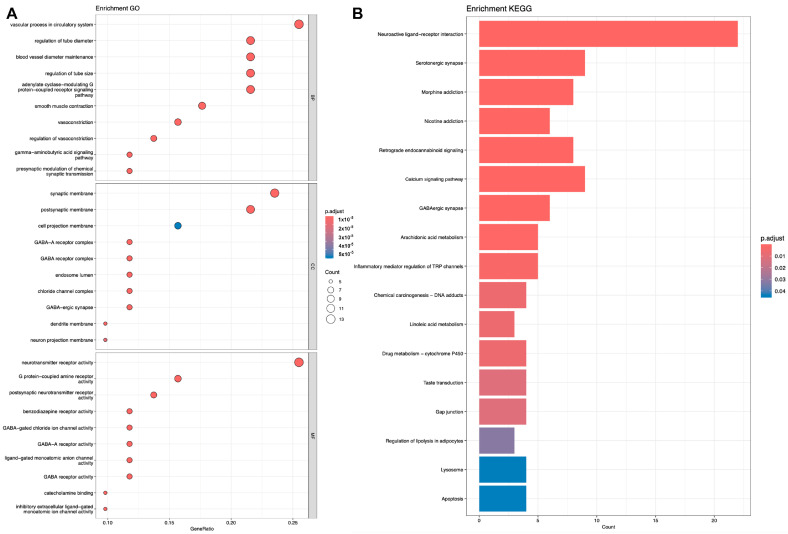
Analysis of representative molecule Dicyclohexyl phthalate. (**A**) GO analysis. (**B**) KEGG analysis.

Subsequently, we performed KEGG [28] and GO [29] analyses on the genes corresponding to the target proteins. Shown in Figure 5, Figure 6 and Figure 7.

The target analysis and KEGG GO analysis revealed the complexity of the molecular mechanisms involved in reproductive toxicity, the diversity of gene pathways, and their differences, indicating that traditional molecule-target research approaches may not be suitable. Therefore, we shifted our focus to studying the intrinsic properties of the molecules.

### 2.3. Machine Learning and Deep Learning

We initially employed various machine learning and deep learning techniques to analyze the molecules, as depicted in Table 1. Among the models evaluated, the DL_Model demonstrated the highest overall performance, with an ACC of 0.88, sensitivity (SE) of 0.83, specificity (SP) of 0.93, and AUC-ROC of 0.88. It also achieved an MCC of 0.77, precision of 0.93, and F1 score of 0.88, reflecting its superior classification quality and predictive accuracy. The SVM_fingerprint model also performed well, achieving an ACC of 0.85, SE of 0.87, SP of 0.83, and AUC-ROC of 0.85. These results indicate that the SVM is particularly effective in identifying reproductive toxic molecules, likely due to its capability of handling high-dimensional data and identifying optimal hyperplanes for classification.

Other models such as Random Forest_fingerprint and KNN_fingerprint showed decent performance, with ACCs of 0.81 and 0.84, respectively. Random Forests (RFs) showed consistent performance across different input types, suggesting their robustness in handling varied molecular data. The Neural Network_fingerprint model also performed reliably, with an ACC of 0.82. However, the Naive Bayes_fingerprint model demonstrated lower performance, with an ACC of 0.75, likely due to its assumption of feature independence, which may not hold true for all molecular descriptors.

Additionally, we compared these models with versions trained on molecular descriptors. The SVM_descriptors model performed similarly to the SVM_fingerprint model, with an ACC of 0.82. The Random Forest_descriptors and Neural Network_descriptors models also showed consistent performance, with an ACC of 0.82 and 0.81, respectively. The Naive Bayes_descriptors model had an ACC of 0.81, which was slightly better than its fingerprint counterpart, but still lower compared to other models.

Overall, the ML_Model outperformed all other models, indicating its robustness and effectiveness in this context. Its high-performance metrics underscore the advantages of using advanced machine learning techniques for predictive modeling in reproductive toxicity. The deep learning model’s superior performance can be attributed to its ability to automatically learn and extract complex features from the data, leveraging both molecular SMILES strings and generated molecular graphs as inputs. This hybrid approach allows the model to capture a wider range of molecular characteristics, leading to improved predictive performance. The significant advantage of using deep learning lies in its capacity to learn intricate patterns and relationships within the data, which traditional machine learning models might not fully capture.

We consulted the literature and tested our models on a validation set, with the specific metrics shown in Table 2. Compared to other models, the deep learning model demonstrated superior overall performance, with a sensitivity of 0.77, specificity of 0.88, and AUC-ROC of 0.91, indicating a strong discriminative ability. The model’s Matthews correlation coefficient (MCC) was 0.65, the accuracy (ACC) was 0.83, and the F1 score was 0.81, reflecting a high classification quality and predictive accuracy. Additionally, the DL_Model outperformed all the other models, including the SVM. In comparison, the Ensemble-Top12 model had a sensitivity of 0.74, specificity of 0.82, AUC-ROC of 0.79, MCC of 0.56, accuracy of 0.78, and F1 score of 0.77, showing slightly lower performance compared to the ML and SVM models. The Rat reproductive toxicity model had a sensitivity of 0.74, specificity of 0.78, AUC-ROC of 0.87, MCC of 0.52, accuracy of 0.76, and F1 score of 0.75, demonstrating similar performance to the Ensemble-Top12 model.

Our deep learning model achieved an impressive accuracy of 0.88 in the test dataset and 0.83 in the validation dataset, surpassing all the machine learning models mentioned earlier. This higher accuracy demonstrates the significant advantages of using hybrid inputs, as the combination of multiple features allows the model to learn more detailed and nuanced information about the molecules. The inclusion of both SMILES strings and molecular graphs enables the deep learning model to capture a wider range of molecular characteristics, leading to improved predictive performance.

However, it is important to note that the slight improvement in accuracy with the deep learning model comes with the disadvantage of increased computational power. Deep learning models typically require more computational resources and memory, which can be a limitation for large-scale applications or in-depth studies. As such, the trade-off between accuracy and computational cost must be carefully considered when choosing the appropriate model for specific applications. Despite this, the superior performance of the deep learning model justifies its use, provided that sufficient computational resources are available.

## 3. Materials and Methods

### 3.1. Data Source

Our research data were sourced from the US Environmental Protection Agency’s Toxicity Reference Database (ToxRefDB) [30], ECHA-C&L Inventory, OECD-eChemPortal [31], and toxic molecules mentioned in reproductive toxicity studies listed on PubMed. Regarding the quality of molecular data involving oral administration to rats, we only included records labeled as “acceptable” and “complete.” We extracted data with the lowest observed adverse effect level (LOAEL) [32] from the “reproductive” category, using them as the standard to determine whether a chemical is toxic to the reproductive system. The molecular structures in the dataset were processed using the Standardiser software package (version 0.1.9, available at https://github.com/flatkinson/standardiser (accessed on 13 May 2024)). This process involved removing counterions, solvent fractions, and salts, followed by the addition of hydrogen atoms, resulting in a standardized set of 1823 small-molecule SMILES data points. The dataset includes 862 positive samples, labeled as 1, and 961 negative samples, labeled as 0.

In this investigation, we applied Morgan fingerprints (ECFP, 2048 bits) to formulate models based on fingerprint patterns, processed using the open-source RDKit platform (version 2023.03.1, http://www.rdkit.org (accessed on 13 May 2024)). Furthermore, we harnessed Mold2 [33] to compute an extensive array of 777 molecular descriptors from two-dimensional (2D) chemical structures. These descriptors are crucial for revealing diverse chemical and physical attributes of molecules and are integral to advancements in drug discovery, chemical research, and environmental studies. The range of descriptors includes atomic and bond counts, functional groups, structural characteristics, autocorrelation, topological indices, and information content indices. Upon generating these descriptors with Mold2, our initial step involved discarding any descriptor that displayed more than 90% zeros across the data, thus maintaining the relevance and variability of our dataset. We then conducted a Shannon entropy [34] analysis to further refine our selection of descriptors. Those with Shannon entropy values below 2.5 were excluded due to their low informational content. The remaining descriptors, characterized by their significant informational value, were subsequently employed as training data for our predictive models.

### 3.2. Chemical Space Distribution of Small Molecules

To analyze the chemical space, molecular fingerprints were computed for each compound using the RDKit [35] library, specifically Morgan fingerprints with a radius of 2, and a bit length of 2048. Each SMILES string was converted into a molecular object to generate the corresponding fingerprint, excluding compounds that could not be converted to valid molecular objects. The two datasets were then combined into a single DataFrame, with a source label added to distinguish between the original datasets. Duplicates based on the SMILES strings were removed to ensure that each compound was unique, and compounds without valid fingerprints were filtered out. The high-dimensional fingerprint data were then subjected to principal component analysis (PCA) [19] to reduce their dimensionality to two principal components, enabling the visualization of the chemical space in two dimensions. The results of the PCA were visualized using a scatter plot, with each point representing a compound in the reduced two-dimensional space.

### 3.3. Clustering Analysis of Small-Molecule Data

To analyze the clustering of small-molecule similarities, we used molecular fingerprints, dimensionality reduction, clustering, and 3D visualization. Morgan fingerprints [36] were computed for each compound using RDKit, with a radius of 2 and 2048 bits. SMILES strings were converted to molecular objects for fingerprint generation, excluding invalid ones. Duplicates were removed to ensure uniqueness in the combined dataset. We applied t-SNE [37] with a perplexity of 30 to reduce the fingerprint data to three dimensions. Hierarchical clustering using Ward’s method was performed on the t-SNE results, setting the number of clusters to 3. Each compound was assigned a cluster label. Within each cluster, we calculated a similarity matrix using the Tanimoto [38] coefficient and identified the molecule with the highest average similarity as the representative. A 3D scatter plot was then created with plotly [39], coloring each compound by cluster assignment and distinctly marking representative molecules, enabling the intuitive exploration of small-molecule similarities and relationships. Next, we conducted a comprehensive evaluation and prediction for the three representative small molecules, using SwissTarget [27] to predict potential targets.

### 3.4. Machine Learning Models

To assess the efficacy of our models, we conducted evaluations on a variety of fundamental machine learning algorithms. The dataset was consistently divided into training, testing, and validation sets in an 8:1:1 ratio to maintain uniformity. The performance of conventional models, trained with Morgan fingerprints and molecular descriptors, was juxtaposed against our customized model, incorporating techniques such as Naive Bayes [40], KNN [41], Random Forest [42], Support Vector Machine [43], and neural networks [44].

Gaussian Naive Bayes: This classifier, based on Bayes’ theorem, is widely utilized for predicting molecular properties and in virtual screening. We optimized the model by tweaking hyperparameters like alpha (from 0.01 to 1) and binarize (at 0, 0.5, or 0.8). Random Forest: Developed by Svetnik et al., this model uses a forest of decision trees, each derived from a bootstrapped sample of compounds and assessing random descriptor subsets. We adjusted several hyperparameters including n_estimators (100, 200, 300), criterion (“gini” or “entropy”), max_depth (5, 10, 20, 30, or None), min_samples_leaf (1 to 10), and max_features (“log2”, “auto”, or “sqrt”). Support Vector Machine: Since its introduction in 1995, SVM has been prominent due to+- its excellent text classification performance. It maximizes class separation by finding the optimal hyperplane. Hyperparameters like the kernel coefficient (gamma at ‘auto’, 0.1-0.2) and the penalty parameter (C at 0.1, 1, 10, 100) were finely adjusted. K-Nearest Neighbors: KNN applies a simple supervised learning algorithm that uses distance metrics (Manhattan, Euclidean, Jaccard) to identify the closest training samples for class prediction. We varied n_neighbors (3, 5, 7, 10, 15, 20) to enhance the model accuracy.

### 3.5. Deep Learning Model Architecture and Feature Transmission

**Figure 8:**
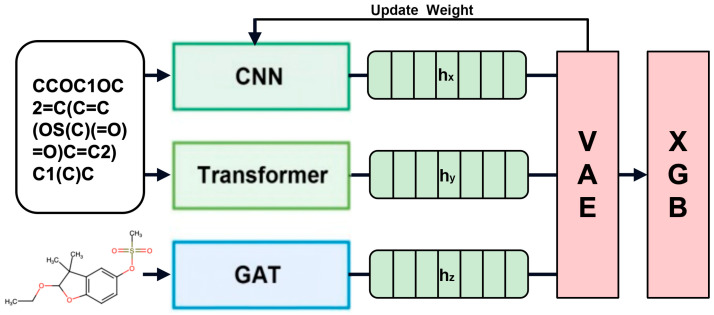
The architecture of the deep learning model.

To analyze the clustering of small-molecule similarities, we developed a comprehensive model architecture that integrates multiple feature extraction techniques. This architecture comprises convolutional neural networks (CNNs) [45], transformers [46], and graph attention networks (GATs) [47] to capture various aspects of molecular data, followed by a Variational Autoencoder (VAE) [48] for dimensionality reduction and feature extraction. In our computational analysis, we employed a range of established libraries renowned for their robustness and efficiency in handling various machine learning tasks. Specifically, we utilized RDKit (version 2023.03.1), accessible at RDKit, for generating molecular fingerprints and preprocessing chemical data. For deep learning implementations, PyTorch (version 2.3.1) provided the backbone, facilitating complex model architectures with its dynamic computation graphs. Graph-based molecular models were managed using DGL (Deep Graph Library, version 2.1.0), which efficiently integrates with PyTorch to enhance operations on graph data. For traditional machine learning algorithms, Scikit-learn (version 1.3.1), a versatile tool, supported our implementations of Support Vector Machine and Naive Bayes classifiers, as well as providing utilities for data splitting and model evaluation (Scikit-learn). The ensemble methods were bolstered by XGBoost (version 2.0.3), chosen for its superior performance in large-scale tree boosting. The model architecture diagram is shown in Figure 8.

CNN for molecular sequences. A CNN was designed to process the SMILES strings. The tokenized sequences were embedded into dense vectors using an embedding layer. Several convolutional layers with ReLU activation functions [49] and max-pooling layers were used to capture local patterns within the sequences. The output from the CNN was a feature vector that encapsulated the local structural information of the SMILES sequences.

Transformer for molecular sequences: To capture long-range dependencies in the SMILES strings, a transformer model was employed. The transformer utilized multi-head self-attention mechanisms to simultaneously focus on different parts of the sequence. Layer normalization and residual connections were incorporated to stabilize the training process and improve convergence. The transformer outputted a feature vector that represented the global context of the SMILES sequences.

GAT for molecular graphs. For molecular graphs, a GAT was implemented. The GAT used attention mechanisms to weigh the importance of neighboring nodes in the graph, thus capturing the most relevant structural information. Multiple attention heads in each layer allowed the model to aggregate information from various perspectives. The output from the GAT was a feature vector that preserved the graph’s structural properties.

The features extracted by the CNN, transformer, and GAT models were concatenated and fed into a VAE for dimensionality reduction and feature extraction [50]. The VAE’s encoder network transformed the concatenated features into a latent space characterized by a mean and log-variance vector. Using a reparameterization trick, latent variables were sampled to ensure differentiability. The decoder network reconstructed the input features from the latent variables, facilitating the learning of robust representations. The VAE was trained to minimize reconstruction loss, thus capturing essential features in a lower-dimensional space.

Model training and feature extraction: The dataset was randomly split into an 8:1:1 ratio for training and validation, ensuring consistency across all models. The CNN model was trained on the tokenized SMILES sequences to capture local patterns. The transformer model, fine-tuned on the SMILES sequences, provided features representing the global context. The GAT model, trained on molecular graphs, captured structural information. The combined features from these models were processed by the VAE, which reduced the dimensionality and extracted salient features. These features were then used to train an XGBoost classifier, leveraging the diverse information captured by the integrated architecture to enhance predictive performance.

## 4. Conclusions

In this study, we undertook a detailed computational analysis of reproductive toxic molecules, identifying three distinct categories: Dimethylhydantoin, Phenol, and Dicyclohexyl phthalate. Our examination encompassed their physicochemical properties, target predictions, and pathways through KEGG and GO analyses. The complex mechanisms identified—ranging from homeostasis regulation disruptions to pathway interferences and membrane protein interactions—underline the sophisticated nature of molecular toxicity.

To navigate these complexities, we employed machine learning and deep learning methodologies to decipher the intrinsic properties of these molecules. Notably, an SVM model using molecular descriptors reached an accuracy of 0.85, while our advanced deep learning model, which incorporates molecular SMILES and generated molecular graphs, surpassed this with an accuracy of 0.88. Both models proved effective in predicting reproductive toxicity, showcasing innovative approaches for toxicity screening that are crucial for pharmaceutical development. Our findings endorse deep learning models as effective tools for reproductive toxicity screening, potentially reducing the reliance on extensive in vivo tests. This approach not only enhances the efficiency of chemical safety evaluations but also uses state-of-the-art modeling techniques to proactively identify high-risk substances, streamlining new chemical development and contributing to significant cost savings.

## Figures and Tables

**Table 1:** Performance metrics of different models in the test dataset.

Model	ACC	SE	SP	MCC	P	F1	BA	AUC
DL_Model	0.88	0.83	0.93	0.77	0.93	0.88	0.88	0.88
SVM_fingerprint	0.85	0.87	0.83	0.71	0.84	0.85	0.85	0.85
Random Forest_fingerprint	0.81	0.80	0.83	0.63	0.83	0.81	0.82	0.81
Naive Bayes_fingerprint	0.75	0.73	0.77	0.50	0.76	0.75	0.75	0.75
KNN_fingerprint	0.84	0.83	0.83	0.67	0.83	0.83	0.83	0.84
Neural Network_fingerprint	0.82	0.80	0.83	0.63	0.83	0.81	0.82	0.82
SVM_descriptors	0.82	0.83	0.80	0.63	0.81	0.82	0.82	0.82
Random Forest_descriptors	0.82	0.80	0.83	0.63	0.83	0.81	0.82	0.82
Naive Bayes_descriptors	0.81	0.77	0.83	0.59	0.82	0.79	0.80	0.81
KNN_descriptors	0.73	0.70	0.77	0.47	0.75	0.72	0.73	0.73
Neural Network_descriptors	0.81	0.80	0.80	0.60	0.80	0.80	0.80	0.81

**Table 2:** Performance metrics of different models in the validation dataset.

Validate	ACC	SE	SP	MCC	P	F1	BA	AUC
DL_Model	0.83	0.77	0.88	0.65	0.87	0.81	0.82	0.91
SVM_fingerprint	0.82	0.74	0.91	0.66	0.89	0.81	0.82	0.88
Ensemble-Top12	0.78	0.74	0.82	0.56	0.81	0.77	0.78	0.79
Rat reproductive toxicity	0.76	0.74	0.78	0.52	0.77	0.75	0.76	0.87

## Data Availability

The original contributions presented in the study are included in the article/Supplementary Materials, further inquiries can be directed to the corresponding author/s.

## Supplementary Materials

The following supporting information can be downloaded at: https://www.mdpi.com/article/10.3390/ijms25147978/s1.
